# Mechanisms of Parkinson’s disease-related proteins in mediating secondary brain damage after cerebral ischemia

**DOI:** 10.1177/0271678X17694186

**Published:** 2017-01-01

**Authors:** TaeHee Kim, Raghu Vemuganti

**Affiliations:** 1 Department of Neurological Surgery, University of Wisconsin, Madison, WI, USA; 2 Neuroscience Training Program, Madison, WI, USA; 3 Cellular & Molecular Pathology Graduate Program, University of Wisconsin, Madison, WI, USA; 4 William S. Middleton Memorial Veterans Administration Hospital, Madison, WI, USA

**Keywords:** Parkinson’s disease, cerebral ischemia, neurodegeneration, neuroprotection, α-synuclein

## Abstract

Both Parkinson’s disease (PD) and stroke are debilitating conditions that result in neuronal death and loss of neurological functions. These two conditions predominantly affect aging populations with the deterioration of the quality of life for the patients themselves and a tremendous burden to families. While the neurodegeneration and symptomology of PD develop chronically over the years, post-stroke neuronal death and dysfunction develop rapidly in days. Despite the discrepancy in the pathophysiological time frame and severity, both conditions share common molecular mechanisms that include oxidative stress, mitochondrial dysfunction, inflammation, endoplasmic reticulum stress, and activation of various cell death pathways (apoptosis/necrosis/autophagy) that synergistically modulate the neuronal death. Emerging evidence indicates that several proteins associated with early-onset familial PD play critical roles in mediating the neuronal death. Importantly, mutations in the genes encoding Parkin, PTEN-induced putative kinase 1 and DJ-1 mediate autosomal recessive forms of PD, whereas mutations in the genes encoding leucine-rich repeat kinase 2 and α-synuclein are responsible for autosomal dominant PD. This review discusses the significance of these proteins with the emphasis on the role of α-synuclein in mediating post-ischemic brain damage.

## Introduction

Chronic neurodegenerative conditions that include Parkinson’s disease (PD), Alzheimer’s disease (AD) and Huntington’s disease (HD) show a progressive and irreversible loss of structure or function of neurons over time with devastating consequences that include a motor to cognitive dysfunction.^1^ Although the pathophysiologic mechanisms responsible for neurodegeneration in chronic disorders are not completely understood, there are many commonalities including oxidative stress, mitochondrial dysfunction, endoplasmic reticulum (ER) stress, inflammation, autophagy, impairment of protein folding and abnormal post-translational modifications leading to overloading of the ubiquitin-proteasome system.^2–5^ Furthermore, several proteins were shown to be associated with the progression of these disorders, particularly those that form aggregates in the CNS over time.^6,7^ For instance, one of the pathological hallmarks of PD is the presence of Lewy Bodies, proteinaceous cytoplasmic inclusions mainly composed of aggregated α-synuclein (α-Syn).^8^ Similarly, AD is manifested by the accumulation of abnormally folded β-amyloid (Aβ) and hyperphosphorylated tau in the brain,^9^ and mutant and aggregated huntingtin proteins that disrupt retrograde transport in neurons play a pivotal role in the pathogenesis of HD.^10,11^ Neurodegeneration associated with acute conditions like stroke and traumatic brain injury (TBI) is also mediated synergistically by oxidative stress, mitochondrial dysfunction, apoptosis, inflammation, and autophagy which play critical roles in its pathogenic progression.^12–14^ This similarity is surprising as the neuronal damage in chronic conditions like PD progresses over years, whereas acute conditions like stroke occur in a short span of hours to days. However, the intensity of mechanisms like oxidative stress, ER stress, and inflammation matches the time of progression as they are at a low, consistent level in chronic disorders, whereas they quickly go to a peak level and dissipate quickly in acute conditions.^15–17^ Interestingly, it was shown that silent strokes could lead to PD via inflammatory responses.^18^ In addition, there is a positive correlation of oligomeric form of PD-causing protein in the red blood cell between ischemic stroke and PD patients.^19^ Furthermore, several proteins involved in the pathogenesis of PD have been reported to play proteopathic roles under the ischemic condition. In this review, we discuss the significance of these proteins with an emphasis on the role of α-Syn in promoting acute brain damage after stroke and the putative mechanisms of action.

## Clinical implications of PD and cerebral ischemia

It has long been suggested that ischemic stroke is associated with PD. For instance, vascular Parkinsonism (VP), a form of atypical Parkinsonism, is produced by one or more small strokes, rather than by the gradual loss of nerve cells as seen in the more typical neurodegenerative PD.^20^ Furthermore, levodopa treatment is known to be less effective and vascular risk factors were more common in VP patients compared to typical PD patients.^21^ Supporting this phenomenon, a recent preclinical study revealed that silent striatal stroke induced by the middle cerebral artery occlusion in rodents, can cause PD and destroys dopaminergic neurons via inflammation in the substantia nigra.^18^ Postmortem studies further raised the possibility of the coexistence of PD and cerebrovascular disease and suggest that patients with Parkinsonism may have cerebrovascular disease without the distinct pathological hallmarks of PD.^22,23^ Earlier clinical studies also reported a higher incidence of ischemic stroke in PD patients compared to control subjects.^24,25^ In particular, the proportion of deaths due to vascular lesions of the brain is significantly higher in male PD patients 45–64 years of age compared with the general population.^24^ Despite such evidence, there is no direct evidence that PD-causing proteins are involved in the pathogenesis of clinical stroke cases. Although levels of oligomeric α-Syn in the red blood cells of ischemic stroke patients were found to be positively correlated with that of PD patients,^19^ prospective studies are needed to clarify the role of PD proteins in human stroke cases.

## The proteopathic basis of neurodegenerative processes in PD and ischemic stroke

PD is the second most common neurodegenerative disease after AD characterized primarily by the loss of dopaminergic neurons in the substantia nigra pars compacta leading to a striatal dopamine deficit. Symptoms of PD include bradykinesia, hypokinesia, rigidity, resting tremor, postural instability, sleep disturbances, depression and cognitive impairment, indicating a more widespread degenerative process.^17^ A pathological hallmark of sporadic PD is the presence of cytoplasmic proteinaceous inclusions called Lewy bodies, mainly composed of α-Syn, ubiquitin, neurofilaments, and molecular chaperones.^26^ The role of Lewy bodies in the disease process and their status as a pathogenic marker of PD is still a matter of discussion. A breakthrough in PD research was the identification of genes which are responsible for monogenic familial forms. Although familial PD with specific genetic defects account for <10% of all cases of PD, the identification of these rare genes and their functions has provided tremendous insight into the pathogenesis of PD and opened up new areas of investigation. Mutations in the genes encoding Parkin, PINK1, and DJ-1 mediate autosomal recessive forms of PD and mutations in the genes encoding leucine-rich repeat kinase 2 (LRRK2) and α-Syn are responsible for autosomal dominant PD. Sporadic and monogenic forms share important clinical, pathological, and biochemical features, notably the progressive demise of dopaminergic neurons in the substantia nigra. Therefore, insight into the function and dysfunction of PD-associated gene products can help elucidate the underlying mechanisms and new pathways in cell death.

Cerebral ischemia triggers a series of complex biochemical and molecular events that promote neuronal death and neurological dysfunction. These include, but not limited to, excitotoxicity, ionic imbalance, edema, oxidative stress, ER stress, and inflammation.^14^ These mechanisms promote necrosis as well as apoptosis and autophagy.^27^ Surprisingly all these mechanisms are also known to be central in promoting brain damage in chronic conditions like PD although the intensity and timing are different between the acute and chronic diseases. Furthermore, recent studies showed that the chronic neurodegeneration-related proteins including α-Syn, DJ-1, Parkin and PINK1 are also involved in the neuronal death following acute insults like a stroke.

### α-Synuclein

#### The structure of α-Syn

**Figure 1.**
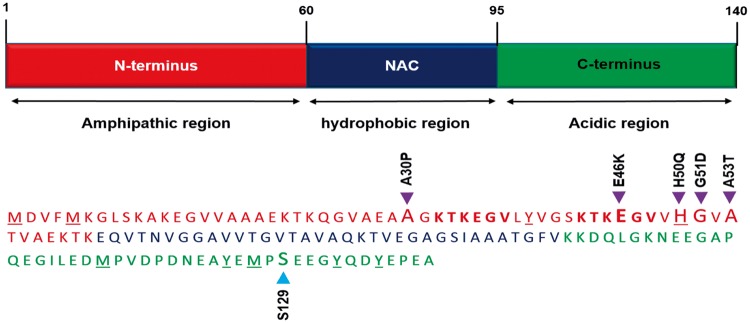
The primary structure of α-Syn. α-Syn monomeric protein consists of an amphipathic N-terminal (amino acids 1–60; Red), a hydrophobic non-amyloid component (NAC; amino acids 61–95; Blue) and an acidic C-terminal (amino acids 96–140; Green). The N-terminal region is highly conserved between the synuclein family proteins and contains characteristic consensus apolipoprotein lipid-binding motifs (KTKEGV). This region adopts an amphipathic α-helical structure upon binding to negatively charged lipids, such as phospholipids present on cellular membranes. The central region is a hydrophobic zone that corresponds to the non-amyloid component and holds the amyloidogenic properties of the protein which allows α-Syn to undergo fibrillization, rich in β-structures. The C-terminal region is a negatively charged acidic segment rich in glutamic, aspartic, and proline residues which hosts several post-translational modification sites including S129. Five missense mutations identified to date (purple) as well as the S129 phosphorylation site (blue) are marked with arrowheads. KTKEGV repeat motifs within the N-terminal are shown in bold. Amino acids associated with nitroxidative modifications (methionine, tyrosine, and histidine) present in either the N-terminal or the C-terminal region are underscored.

α-Syn is a small protein (14.5 kDa; 140 amino acid) abundantly expressed in the mammalian brain and localized primarily in presynaptic terminals. α-Syn has three structural domains: the amphipathic N-terminal region (residues 1 to 60), the central hydrophobic region (residues 61 to 95; also known as NAC; non-amyloid component) and the acidic C-terminal region (residues 96 to 140) (Figure 1). The N-terminal region is well conserved and binds to phospholipids to acquire an amphipathic α-helix structure.^28^ The central hydrophobic region enables α-Syn to form aggregates, rich in β-pleated sheets upon aggregation. The tertiary interactions between the three domains are known to stabilize and shield the NAC region from undergoing spontaneous oligomerization of α-Syn.^29,30^ At least five missense mutations in the N-terminal and several post-translational modifications including nitroxidation, phosphorylation, and ubiquitination in both the N- and C-terminals are implicated in the pathogenesis of PD and other synucleinopathies as they disrupt the intrinsic autoinhibitory mechanisms leading to oligomerization of α-Syn.^31–35^

#### Oligomeric α-Syn and the cellular toxicity

**Figure 2.**
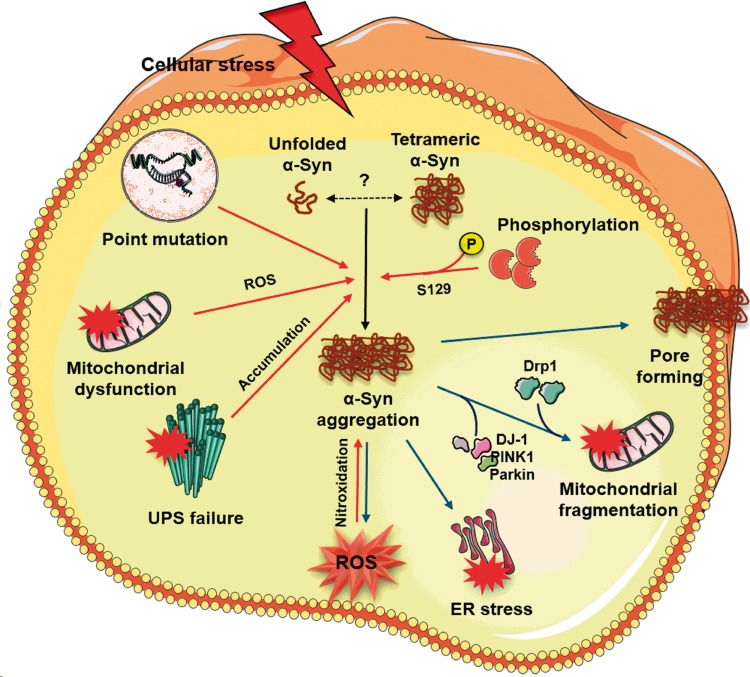
Mechanism of oligomeric α-Syn-mediated cellular toxicity in PD. In a normal physiological condition, α-Syn has long been known to exist as an unfolded monomer. However, recent evidence suggests that α-Syn under normal physiological state may exist as tetramers that resist aggregation. Tetrameric α-Syn species become destabilized and prone to aggregation due to several pathologic factors including point mutations, ROS generated by dysfunctional mitochondria and post-translational modifications like phosphorylation and nitroxidation. Concomitantly, impaired ubiquitin-proteasomal system (UPS) may not effectively clear the misfolded proteins resulting in the accumulation of oligomeric α-Syn species. α-Syn aggregates, in turn, increase cellular toxicity by several mechanisms. The α-Syn oligomers (1) permeabilize the lipid bilayer by forming Ca^2+^-permeable pore-like complexes thereby disturbing intracellular calcium homeostasis, (2) promote mitochondrial fragmentation by directly interacting with mitochondrial complex I, recruiting mitochondrial fission protein Drp1 and inhibiting DJ-1/PINK1/Parkin proteins that are known to maintain mitochondrial integrity, (3) activate the prolonged unfolded protein response (UPR) by accumulating within the ER lumen, and (4) generate mitochondria-independent ROS via redox activities with metal ions.

α-Syn aggregates, the major component of Lewy bodies, are implicated in the cytotoxicity and the pathogenesis of several neurodegenerative disorders.^36–38^ However, soluble oligomeric intermediates are considered as the major precipitators of PD pathology as mature fibrils are deemed benign precipitates by sequestration of toxic oligomers.^39–41^ Biochemical and genetic studies suggest that α-Syn directly interacts with a number of key players in the pathogenesis of synucleinopathies (Figure 2). α-Syn oligomers are thought to form annular structures and pore-like complexes in the lipid bilayer of the membranes resulting in disruption of calcium homeostasis.^41–45^ However, α-Syn oligomers were also thought to induce membrane permeabilization independent of pore-like complexes.^46,47^ α-Syn can also promote mitochondria-independent reactive oxygen species (ROS) production, by interacting with metal ions. In vitro studies showed that α-Syn has several binding sites for copper and the redox activities of α-Syn-Cu^+^ complexes can generate hydrogen peroxide,^48,49^ and these α-Syn-copper complexes are found to be neurotoxic and promote further oligomerization.^50,51^ The unfolded protein response (UPR) and the subsequent ER stress are neuroprotective in moderation. However, prolonged and/or excessive ER stress leads to neuronal death.^52^ In the brain of PD patients, expression of UPR activation markers, phospho-PERK and phospho-eIF2α, was observed to be increased and colocalized with α-Syn.^53^ An in vivo study further showed that toxic α-Syn oligomers accumulate within ER lumen and α-Syn oligomer-dependent ER stress can be rescued by treatment with Salubrinal, which inhibits eIF2α and thus decreases protein overload^54,55^. α-Syn oligomers are also shown to be promote inflammatory responses^56,57^ and autophagy.^58,59^

#### α-Syn and mitochondrial dysfunction

Mitochondrial homeostasis are dependent on fusion and fission dynamics, and their perturbation results in mitochondrial dysfunction which is implicated in neurodegenerative disorders.^60^ In the brains of PD patients, aggregated α-Syn was observed to be localized in the inner mitochondrial membranes where it interacts with complex I resulting in the reduced mitochondrial complex I activity and increased ROS production.^61–63^ α-Syn aggregates also interact with other mitochondrial proteins such as carbonic anhydrase, enolase, and lactate dehydrogenase which were found to be oxidized in brains of transgenic mice overexpressing mutant A30P α-Syn.^64^ α-Syn overexpression was shown to significantly increase the translocation of mitochondrial fission protein Drp1 via extracellular signal-regulated kinase (ERK) pathway^65^ and induced mitochondrial cytochrome C release and caspase-9 and -3 activities promoting apoptosis.^55,66^ In contrast, inhibition of α-Syn expression prevented MPP^+^-induced mitochondrial fragmentation in vitro.^67^ As mitochondrial dysfunction is thought to be a key player in neurodegeneration, these studies suggest that α-Syn could potentially play a pathogenic role in a broad spectrum of neurodegerative diseases.

#### α-Syn and post-translational modifications

As discussed above, post-translational modifications are known to impact the pathological role of α-Syn including its aggregation, fibrillation, Lewy body formation and neurotoxicity. Of various post-translational modifications α-Syn can undergo, serine-129 (S129) phosphorylation is considered as a defining hallmark of PD and other synucleinopathies.^68,69^ In the post-mortem PD patient brains, > 90% of the α-Syn aggregates are known to be S129 phosphorylated, but its functional significance is not yet completely elucidated.^70^ Many kinases can promote S129 phosphorylation of α-Syn, and overexpression of GRK2 and GRK6 in Drosophila^71^ and rodent PD models^72^ showed enhanced and accelerated neuronal death. In contrast, overexpression of Polo-like kinase 2 (PLK2) which also mediates α-Syn S129 phosphorylation was shown to reduce α-Syn-associated dopaminergic neuronal loss and ameliorate hemiparkinsonian motor impairment presumably by enhancing autophagy in adult rodents.^73^ Although further studies are required for elucidating the exact function of S129 phosphorylation on α-Syn toxicity, these studies emphasize the significance of the kinase responsible for the phosphorylation rather than the phosphorylation itself.

#### α-Syn: Unanswered questions

Although extensive research over the last two decades has provided tremendous insight into the role of α-Syn in the pathogenesis of PD, there are still many unanswered questions that need to be addressed for developing therapies targeting α-Syn. While the predominant view is that α-Syn exists as an unfolded monomer under native conditions, recent studies showed that α-Syn may exist as a helically folded tetramer that resists aggregation and fibrillation in the normal brain.^74,75^ Neurons derived from PD patients expressing A53T mutant α-Syn contained decreased tetramers.^76^ In vitro studies further showed that α-Syn missense mutations (A30P, E46K, and A53T) lower α-Syn tetramer: monomer ratio and induce neurotoxicity and round inclusions.^76^ In addition, mutagenesis studies revealed that the α-Syn KTKEGV repeat motifs present in the N-terminus mediate physiological tetramerization and perturbing them causes PD-like neurotoxicity.^77^ Despite these studies, it is not convincingly established if α-Syn exists as a monomer or a tetramer under normal conditions.^78,79^

Another enigma regarding α-Syn pathology is the mechanisms that modulate α-Syn expression during pathological states. Interestingly, microRNAs miRNA-7 and miRNA-153 were shown to synergistically target α-Syn mRNA to repress its protein expression.^80,81^ In human embryonic kidney cells (HEK293T) challenged with hydrogen peroxide, premiR-7 transfection decreased α-Syn protein expression and cell death.^81^ In addition to direct effects of miRNAs on the α-Syn protein expression, these small non-coding RNAs may also have indirect implications for the α-Syn pathology in a specific cell type. For example, miR-155 knockout mice showed reduced microgliosis and neuroprotection despite α-Syn overexpression.^82^ miR-155 was also observed to be essential for α-Syn-induced major histocompatibility complex class II (MHCII) and inducible nitric oxide synthase (iNOS) expression in microglia.^82^ Although further studies are needed, these findings not only provide mechanisms by which α-Syn levels are regulated in disease brains but also raise the possibility that miRNA based therapy is feasible for α-Synucleinopathies.

#### α-synuclein and stroke

Interestingly, cellular environment following cerebral ischemia that includes inflammation, oxidative stress and ER stress potentially provides optimal conditions for α-Syn aggregation. In PD and other chronic neurodegenerative conditions, α-Syn aggregates exacerbate cellular toxicity by interacting with lipid membranes,^41^ metal ions,^48^ ER,^54^ and mitochondria.^63^ Stroke can also either initiate or accelerate progressive neurodegenerative processes and is a known epidemiologic risk factor for AD.^83^ Significance of α-Syn in modulating ischemic brain injury was demonstrated by multiple labs (Table 1). Ishimaru et al.^84^ and Polymeropoulos et al.^85^showed that a brief global ischemia in gerbils resulted in increased α-Syn (*aka* precursor protein of non-Aβ component of AD amyloid; NACP) immunoreactivity in the hippocampus where neurons died and proposed that NACP may be related to several neurodegenerative conditions. Subsequently, Hu et al.^86^ showed that hypoxia/ischemia increases α-Syn protein levels in the rat cerebral cortex, and Yoon et al.^87^ reported increased α-Syn immunoreactivity and protein levels in the hippocampal CA1 region following global ischemia in gerbils and this process was more prominent in aged than young animals.^87^ More importantly, increased α-Syn expression and neuronal death were attenuated by treatment with the antioxidant enzyme SOD1, indicating that ROS promotes α-syn expression and aggregation.^87^ Furthermore, global cerebral ischemia in α-Syn knockout mice resulted in increased prostaglandin levels indicating the role of α-Syn in mediating post-ischemic inflammatory responses.^88^ This study suggested that α-Syn induction after ischemia may be neuroprotective by reducing the post-ischemic brain prostaglandin formation, but later studies challenged this notion. Although prostaglandin is implicated in both pro-inflammatory and anti-inflammatory responses in different biologic systems,^89^ a recent in vivo study showed that stimulation of prostaglandin D2 receptor DP1 is neuroprotective in the rodent ischemic brain.^90^ Furthermore, Unal-Cevik et al.^91^ reported that a brief ischemic insult is sufficient to induce α-Syn aggregation in neurons in adult mice. When subjected to a brief ischemia, α-Syn overexpressing transgenic mice showed exacerbated infarction and enhanced 3-nitrotyrosine immunoreactivity, indicating oxidative/nitrative stress as a potential mechanism of α-Syn-mediated toxicity similar to what was shown in PD. Recent studies from our laboratory also conclusively demonstrated that α-Syn induced after transient focal ischemia plays a detrimental role.^92^ We observed that transient focal ischemia induced by MCAO in adult rats significantly upregulates α-Syn mRNA and protein levels, and knockdown of α-Syn decreases infarction and promotes better neurobehavioral recovery. Knockdown of α-Syn also attenuated known ischemic pathological markers including mitochondrial dysfunction (Drp1), apoptosis (cleaved caspase-3), autophagy (LC-3 II/I ratio) as well as oxidative stress (3-nitrotyrosine). In addition, we observed that α-Syn protein oligomerizes, aggregates, and translocates to neuronal nuclei in both rat and human post-stroke brain. α-Syn knockout mice showed curtailed mortality, smaller infarcts, and better neurological recovery when subjected to transient focal ischemia. Furthermore, we observed that phosphorylation of α-Syn is responsible in part for its toxicity in the post-stroke brain as mice that lack PLK2 (the predominant kinase that mediates S129 phosphorylation of α-Syn) showed better functional recovery and smaller infarct after transient focal ischemia.^92^ These studies show that α-Syn plays a critical role even in the pathogenesis of ischemic stroke. Prospective studies will elucidate the exact mechanisms of α-Syn-mediated ischemic cell death.

**Table 1.** Role of α-Syn in cerebral ischemia.

Model	Duration	Age/Sex	Species	Expression	Observations	Ref.
Global ischemia	5 min	YA; M	Gerbil	upregulated	↑ α-Syn around blood vessels in CA1 on day 4; ↑ α-Syn^+^ tubal structures on month 6	^84^
HI	90 min	P7; F	Rat	upregulated	2-fold increase 2 h after HI	^86^
Global ischemia	5 min	6 m, 24 m; M	Gerbil	upregulated	↑ α-Syn in CA1 in aged gerbil; SOD1 inhibited α-Syn	^87^
Global ischemia	30 sec	9–11 m; M	Mouse	N/A	α-Syn^−/−^ increased brain PG formation following ischemia	^88^
tMCAO	30 min	8w; M	Mouse	upregulated	Brief ischemia induced α-Syn aggregation, worsened damage	^91^
tMCAO	60–90 min	YA; M	Rat, Mouse	upregulated	↓ α-Syn reduced infarcts and improved neurological recovery	^92^

YA: young adults; M: male; F: female; P: postnatal; m: months; w: weeks; HI: hypoxia/ischemia; tMCAO: transient middle cerebral artery occlusion; PG: prostaglandin; ↑: increase; ↓: decrease.

### Parkin

Parkin is a widely expressed cytosolic ubiquitin E3 ligase in the brain that mediates mono- and polyubiquitination of many proteins to regulate a variety of cellular processes. A number of proteins identified as Parkin substrates are implicated in the pathogenesis of PD. Among those, Parkin-mediated degradation of the GTPase cell division control-related protein-1 (CDCrel-1), is known to induce dopaminergic neurodegeneration in the rodent brain.^93,94^ In addition, overexpression of Parkin-associated endothelin receptor-like receptor (Pael-R) induces the unfolded stress response in dopaminergic neurons, and Parkin attenuates insoluble Pael-R-mediated cellular toxicity presumably through ubiquitination.^95^ Furthermore, the α-Syn-interacting protein, synphilin-1, interacts with and is polyubiquitinated by Parkin leading to the formation of protein aggregates when overexpressed together with α-Syn.^96^ Recent studies suggest that lysine-63-mediated ubiquitination on synphilin-1 may participate in the degradation of Lewy body inclusion independent of the UPS, implicating its role in the inclusion formation.^97^ Overall, the catalytic activities of Parkin were shown to be neuroprotective and counters diverse cellular insults including α-Syn toxicity and UPS dysfunction,^98^ accumulation of Pael-R (a substrate of Parkin),^95^ and kainite-induced excitotoxicity.^99^

Parkin gene is a common cause of monogenic PD, and Parkin can undergo > 100 types of mutations including exonic rearrangements, point mutations, and small deletions or insertions.^100^ Furthermore, Parkin can be inactivated by post-translational modifications due to oxidative and nitrosative stress as seen in sporadic PD.^101,102^ Parkin inactivation leads to accumulation of Parkin substrates like aminoacyl-tRNA synthetase-interacting multifunctional protein type 2 (AIMP2) and far upstream element-binding protein 1 (FBP1) which leads to neurodegeneration in PD.^103,104^ The nonreceptor tyrosine kinase c-Abl phosphorylates tyr-143 and thus inhibits ubiquitin E3 ligase activity of Parkin leading to neuronal degeneration in rodent MPTP model.^105^ Concomitantly, activated c-Abl further contributes to α-Syn-induced neurodegeneration in the rodent brain.^106^ Parkin inactivation also leads to induction of its substrates PARIS (ZNF746) and RTP801/REDD1, which promote dopaminergic neurodegeneration. Once accumulated, PARIS represses the peroxisome proliferator-activated receptor gamma-coactivator 1-alpha (PGC-1α) and its downstream neuroprotective target gene NRF-1,^107^ while RTP801/REDD1 is a negative regulator of survival kinases mTOR and Akt and hence promotes apoptosis.^108^ Taken together, these studies indicate that Parkin is an essential neuroprotective protein, and its inactivation promotes neurodegeneration.

## PINK1

PTEN-induced kinase 1 (PINK1) is a mitochondrial serine-threonine kinase that is ubiquitously expressed throughout the brain and is found in most cell types.^109^ More than 40 mutations in PINK1 gene are known to promote autosomal recessive PD.^110,111^ Several lines of evidence indicate that PINK1 is also a neuroprotective protein. An early study showed that wild-type, but not mutant PINK1 protects SH-SY5Y cells against stress-induced mitochondrial dysfunction and apoptotic cell death.^42^ Depletion of PINK1 both in vitro and in vivo was shown to exacerbate α-Syn aggregation-mediated toxicity as well as promote proteasomal inhibition.^112,113^ In contrast, reinstating PINK1 in Drosophila can rescue α-Syn-induced pathologic phenotypes although the mechanisms of how PINK1 mitigating α-Syn toxicity are not clearly known.^114^ PINK1 colocalizes and phosphorylates TRAP1 (TNF receptor-associated protein 1; HSP75), which is a protein chaperone that protects mitochondria from oxidative stress.^115^ In Drosophila*,* TRAP1 overexpression was shown to rescue PINK1 loss-of-function phenotypes.^116^

Much attention is given to the role of PINK1 in conjunction with Parkin in promoting the clearance of impaired mitochondria. Termed mitophagy, it is the highly selective autophagy-based process representing a critical mechanism to eliminate damaged and dysfunctional mitochondria, thus protecting cells from aging, oxidative stress, and even apoptotic cell death.^117^ When mitochondria are damaged, mitochondrial membrane depolarization leads to PINK1 accumulation on the outer membrane, which recruits Parkin from cytosol that ubiquitinates mitofusin 1, mitofusin 2 and P62/SQSTM1 that promotes mitophagy to recycle the healthy mitochondrial components.^117–124^ A recent study suggested that PINK1 can also promote mitophagy without involving Parkin by directly phosphorylating autophagy receptors NDP52 and optineurin which in turn recruit the autophagy factors ULK1, DFCP1, WIPI1 as well as LC3.^125^ This evidence indicates that PINK1 and Parkin, the two genes implicated in the autosomal recessive PD, work together in the same pathway to govern mitochondrial quality control, bolstering previous evidence that mitochondrial damage is involved in PD.

Similar to their role in PD and other neurodegenerative disorders, even after stroke, Parkin and PINK1 are thought to regulate the mitochondrial integrity and thereby protect neurons from stress-induced mitochondrial dysfunction.^126–128^ Transient focal ischemia in adult mice was shown to markedly downregulate Parkin protein expression within the first 24 h of reperfusion.^129^ Overexpression of Parkin in primary cortical neurons further showed that neurons were protected from injury induced by ER stress which is a known mediator of post-ischemic secondary brain damage.^129^ On the contrary, treatment of mice subjected to transient focal ischemia with ER stress inducers tunicamycin and thapsigargin prevented post-ischemic Parkin depletion leading to its translocation to mitochondria and activation of mitophagy.^130,131^ In addition, the neuroprotective effects of these ER stress inducers were reversed by Parkin knockdown and/or knockout.^130,131^ Surprisingly, recent studies showed that Parkin and PINK1 were upregulated in cortical penumbra following photothrombotic focal ischemia in rats and promote the survival of penumbral neurons.^132^ ^133^ Moreover, Parkin protein upregulation was also shown in the rat brain after transient focal cerebral ischemia induced by MCAO.^134^ Thus, it is still premature to conclude the definitive role of Parkin in ischemic brain damage.

## DJ-1

DJ-1 is a small ubiquitously expressed protein in the brain that exists as a homodimer in cytoplasm, mitochondria, and nucleus.^135–137^ DJ-1 is an oxidative stress response protein that defends cells against ROS.^138–140^ DJ-1 is thought to help maintain the integrity of mitochondria, presumably by interacting with complex I, and thereby minimizing the production of mitochondria-dependent oxidative stress leading to cytoprotection.^141^ Loss of DJ-1 leads to pathological mitochondrial phenotypes including reduced membrane potential,^142^ increased fragmentation,^143,144^ and accumulation of autophagic markers.^145^

Studies showed that brains of AD and PD patients contain high levels of oxidized DJ-1 which is thought to be neuroprotective.^146,147^ The neuroprotective potential of DJ-1 is considered to be due to post-translational modifications including oxidation of cysteine C106,^148–152^ SUMOylation of lysine K130 ^153,154^ and transnitrosylation of cysteine residues C46, C53, and C106.^155^ Especially, from the transnitrosylated C106 residue, the NO group can be transferred to phosphatase and tensin homolog (PTEN) that decreases its phosphatase activity leading to neuronal survival.^156^ Although DJ-1 directly quenches some ROS via oxidation of Cysteine residues, this effect is not deemed a major contributor to the protective function of DJ-1.^157^ The neuroprotective activity of DJ-1 is thought to be multifactorial that includes chaperoning misfolded proteins including α-Syn and controlling its aggregation,^158–160^ activating SOD1^161^ and modulating transcriptional regulators like p53,^162,163^ nuclear factor erythroid 2-related factor 2 (Nrf2)^164,165^ and protein-associated splicing factor (PSF)^166^ as well as by acting as a cysteine protease.^167,168^ DJ-1 acts in parallel with PINK1/Parkin to protect mitochondria during oxidative stress. Overexpression of Parkin or PINK1 prevents rotenone-induced mitochondrial fragmentation in the absence of DJ-1 in human dopaminergic cells.^145^ On the contrary, DJ-1 overexpression ameliorates PINK1, but not Parkin mutant-mediated mitochondrial dysfunction in Drosophila.^169^ Furthermore, mitochondrial fragmentation induced by α-syn overexpression in C. Elegans was shown to be mitigated by coexpression of wild-type PINK1, Parkin or DJ-1 with α-Syn but not by mutant forms of PINK1, Parkin or DJ-1.^170^

DJ-1 is the most studied PD-causing protein in ischemic stroke setting due to its known neuroprotective roles in PD and other neurodegenerative conditions as an antioxidant as well as redox-sensitive molecular chaperone. Consistent with previous findings in PD, several studies showed that focal ischemia in rodents induces DJ-1 protein expression which prevents ischemic neuronal death by suppressing ROS production and acting as a redox-sensitive molecular chaperone.^134,171–174^ Biochemical and immunocytochemical studies revealed that when rat primary neuronal cultures were subjected to oxygen-glucose deprivation, DJ-1 translocates to mitochondria and nucleus which was thought to protect the cells.^175,176^ Interestingly, the translocated mitochondrial DJ-1 was subsequently observed to be secreted into the medium which is an essential step in DJ-1-mediated neuroprotection.^175,176^ In vivo and in vitro studies also showed the significance of astrocytic DJ-1 in response to post-ischemic oxidative stress.^177^ Following focal ischemia, activated astrocytes induced DJ-1 expression in the rat brain. Furthermore, when primary astrocytic cells were treated with H_2_O_2_, DJ-1 was observed to be secreted from astrocytes and scavenged hydroxyl radicals. However, the functional significance DJ-1 secretion is not clear yet.

**Figure 3.**
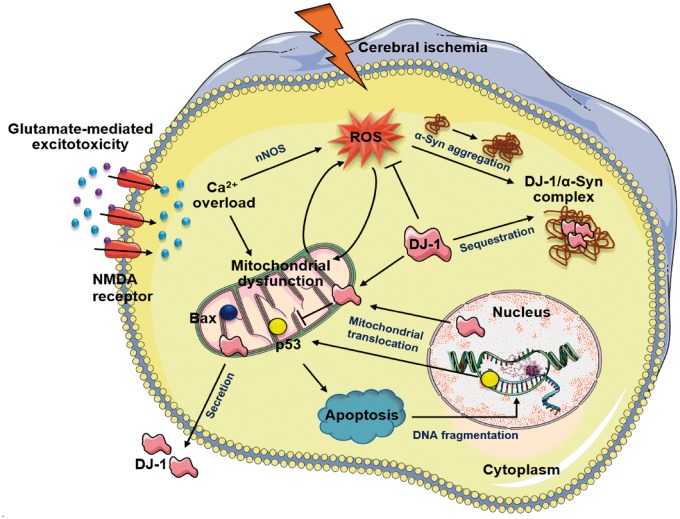
Proposed neuroprotective role of DJ-1 following cerebral ischemia. During the ischemic cascade, glutamate-induced excitotoxic stimulation of Ca^2+^-permeable NMDA receptors and subsequent Ca^2+^ influx activates intracellular Ca^2+^ sensitive enzymes such as nNOS. Oxidative stress produced by ROS damages organelles including mitochondria and promotes aggregation of α-Syn. Following ischemic insult, DJ-1 translocates into neuronal mitochondria and nuclei to act as an antioxidant and molecular chaperone. Once translocated, DJ-1 negatively regulates proapoptotic factors like p53 and Bax and inhibits apoptotic cell death. DJ-1 is also secreted into the extracellular space, but its functional significance is not yet clear. Upon ischemic stress, aggregated α-Syn may form higher molecular weight complexes with DJ-1 resulting in decreased solubility and bioavailability of DJ-1.

Judging from previous evidence from PD and other neurodegeneration models, α-Syn aggregates might prevent the neuroprotective potential of DJ-1 after ischemia (Figure 3). Brain tissue from PD patients was shown to contain large molecular complexes (>2000 kDa) of α-Syn and DJ-1.^178,179^ In addition, decreased DJ-1 solubility was observed in the brains of PD patients.^178,180^ These studies suggest that α-Syn aggregates recruit DJ-1 to form insoluble inclusions and thereby attenuate its antioxidant neuroprotective ability. DJ-1 may also exert cellular protection by regulating transcription factors involved in the cell death. In support to this notion, DJ-1 was shown to negatively regulate p53 in response to oxidative stress.^154,162^ In zebrafish model of PD, DJ-1 deficiency resulted in increased p53 and Bax (apoptosis regulator) expression when challenged by H_2_O_2_.^181^ Activation of p53 is also involved in apoptotic cell death in cerebral ischemia and in vivo administration of p53 inhibitor pifithrin-α induced neuroprotection in rats subjected to focal ischemia.^182–184^ Taken together, it could be hypothesized that ischemic stress-induced α-Syn accumulation contributes, at least in part, to the increased p53 levels via suppressing DJ-1 solubility.

## LRRK2

Leucine-rich repeat kinase 2 (LRRK2) is a multidomain protein (280 kDa, 2527 amino acid) which contains a central catalytic core that includes a Ras of complex (ROC), a GTPase protein domain, a carboxy-terminal of Roc (COR) domain and a kinase domain. The central catalytic core is surrounded by several potential protein–protein interacting domains including leucine-rich repeats and the WD40 domain.^185^ Mutations in the LRRK2 (R1441C, R1441G, and R1441H in the ROC domain, G2019S and I2020T in the kinase domain and Y1699C in the COR domain) are thought to be responsible for both familial and sporadic PD.^186^ Although the exact functional consequences of various LRRK2 mutations are not known, mutations in the ROC and COR domains decrease GTPase activity while mutations in the kinase domain increases kinase activity.^187–189^ Among the known LRRK2 mutations, G2019S mutation in the kinase domain shows the most prevalence in PD population.^190,191^ As G2019S mutation is the most prevalent in the PD population, increased LRRK2 kinase activity by G2019S mutation implicates significance in the pathogenesis of monogenic PD. In addition, polymorphism within the LRRK2 gene is also correlated with sporadic PD risk.^192,193^ Many studies further showed that LRRK2 variants that include A419V, R1628P, and G2385R mutations increase risk for sporadic PD in Asians.^194–196^ Interestingly, there might be an association between LRRK2 and other PD genes; and many PD patients with LRRK2 mutations exhibit α-Syn-positive Lewy bodies and tau-related pathology.^197,198^

LRRK2 is known to phosphorylate Tau directly or indirectly by activating GSK-3β which is a predominant Tau kinase.^199–201^ Tau is a microtubule-associated protein that is predominantly expressed in CNS and regulates neurite outgrowth and axonal transport by modulating tubulin assembly and microtubule stability.^202^ Hence, proper functioning of Tau is essential for neuronal plasticity and memory consolidation. The microtubule-binding ability of Tau is regulated by several post-translational modifications. Particularly, hyperphosphorylation of Tau promotes neuronal degeneration, and the fibrillar deposits of highly phosphorylated Tau is a defining marker of neurodegeneration including.^203^ Studies in PD and AD models showed co-localization of α-Syn with phospho-GSK-3β and phospho-Tau, and indicated that α-Syn may act as a scaffolding molecule that is necessary for the activation of GSK-3β and the subsequent Tau hyperphosphorylation.^204,205^ Taken together, these studies indicate that LRRK2 may be critically involved in the Tau pathology in PD.

**Figure 4.**
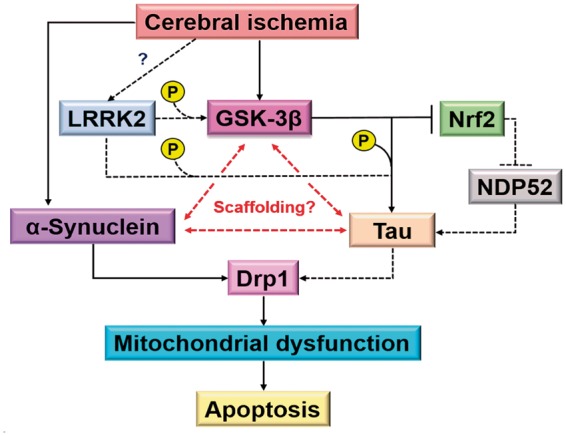
LRRK2-mediated apoptotic cell death in cerebral ischemia. Although mutations in Tau is not known to cause monogenic PD, fibrillar deposits of highly phosphorylated Tau is a defining marker of several neurodegenerative diseases and is also implicated in apoptosis. LRRK2 was shown to mediate Tau phosphorylation. LRRK2 also phosphorylates GSK-3β which in turn phosphorylates Tau and concomitantly suppresses Nrf2 that inhibits Tau phosphorylation via induction of NDP52. α-Syn co-localizes with phospho-GSK-3β and phospho-Tau, suggesting that α-Syn may act as a scaffolding molecule as well as necessary for the activation of GSK-3β and the subsequent Tau hyperphosphorylation. α-Syn is shown to be rapidly induced following ischemia and suppression of α-Syn expression attenuates mitochondrial fragmentation. In addition, phospho-Tau colocalizes with TUNEL staining and interacts with Drp1 in neurons. Dotted line shows changes reported in chronic neurodegenerative conditions and the solid line shows changes reported in cerebral ischemia.

As indicated above, LRRK2 activates GSK-3β by phosphorylation leading to tau hyperphosphorylation in PD.^204,205^ In parallel to this, cerebral ischemia in rodents was shown to increase GSK-3β-mediated tau hyperphosphorylation that colocalizes with TUNEL staining implicating its role in apoptotic cell death.^206,207^ Concomitantly, GSK-3β downregulates Nrf2 following cerebral ischemia,^208^ which is known to reduce levels of hyperphosphorylated tau protein by inducing autophagy adaptor protein NDP52.^209^ Furthermore, α-Syn was shown to be induced following cerebral ischemia and its knockdown decreases post-ischemic mitochondrial fragmentation and apoptosis.^92^ Thus, LRRK2 might contribute to post-ischemic apoptotic cell death as well by modulating tau phosphorylation in the presence of α-Syn (Figure 4). Future studies will show the functional significance of LRRK2 to post-stroke brain damage.

## Conclusions

The pathophysiologic mechanisms of neuronal death and therapeutic development for chronic neurodegenerative diseases like PD and acute injuries like stroke are usually investigated separately. However, many molecular mechanisms that promote secondary brain injury like inflammation, oxidative stress, ER stress, apoptosis, and autophagy can be seen in both chronic and acute CNS insults although their intensity and timing are different. Furthermore, observations like aggregation of α-Syn and depletion of proteins like DJ-1, PINK1, and Parkin after cerebral ischemia suggest that the mechanisms that are known to promote pathology in chronic conditions might also contribute to neuronal death after stroke. However, the causal relationship between the chronic neurodegeneration-associated proteins and cell death in stroke remains to be established beyond doubt. It is possible that neuronal cell injury may be caused by a direct toxic effect of protein aggregates or protein depletion. Conversely, abnormal protein levels could arise due to the pathological cellular environment created by injured neurons. Future studies are warranted to elucidate the molecular mechanisms underlying pathological processes mediated by mutual key proteins.

## References

[1] Sheikh S Safia Haque E Neurodegenerative diseases: Multifactorial conformational diseases and their therapeutic interventions. J Neurodegener Dis 2013; 2013: 563481. 26316993 10.1155/2013/563481 PMC4437348

[2] Nixon RA The role of autophagy in neurodegenerative disease. Nat Med 2013; 19: 983–997. 23921753 10.1038/nm.3232

[3] Barnham KJ Masters CL Bush AI Neurodegenerative diseases and oxidative stress. Nat Rev Drug Discov 2004; 3: 205–214. 15031734 10.1038/nrd1330

[4] Petrucelli L Dawson TM Mechanism of neurodegenerative disease: Role of the ubiquitin proteasome system. Ann Med 2004; 36: 315–320. 15224658 10.1080/07853890410031948

[5] Beal MF Mitochondrial dysfunction in neurodegenerative diseases. Biochim Biophys Acta 1998; 1366: 211–223. 9714810 10.1016/s0005-2728(98)00114-5

[6] Takalo M Salminen A Soininen H Protein aggregation and degradation mechanisms in neurodegenerative diseases. Am J Neurodegener Dis 2013; 2: 1–14. 23516262 PMC3601466

[7] Soto C Unfolding the role of protein misfolding in neurodegenerative diseases. Nat Rev Neurosci 2003; 4: 49–60. 12511861 10.1038/nrn1007

[8] Kim WS Kagedal K Halliday GM Alpha-synuclein biology in Lewy body diseases. Alzheimers Res Ther 2014; 6: 73. 25580161 10.1186/s13195-014-0073-2 PMC4288216

[9] Bloom GS Amyloid-beta and tau: The trigger and bullet in Alzheimer disease pathogenesis. JAMA Neurol 2014; 71: 505–508. 24493463 10.1001/jamaneurol.2013.5847 PMC12908160

[10] Liot G Zala D Pla P Mutant Huntingtin alters retrograde transport of TrkB receptors in striatal dendrites. J Neurosci 2013; 33: 6298–6309. 23575829 10.1523/JNEUROSCI.2033-12.2013 PMC6619069

[11] Gharami K Xie Y An JJ Brain-derived neurotrophic factor over-expression in the forebrain ameliorates Huntington's disease phenotypes in mice. J Neurochem 2008; 105: 369–379. 18086127 10.1111/j.1471-4159.2007.05137.x PMC2377033

[12] Nakka VP Prakash-Babu P Vemuganti R Crosstalk between endoplasmic reticulum stress, oxidative stress, and autophagy: Potential therapeutic targets for acute CNS injuries. Mol Neurobiol 2016; 53: 532–544. 25482050 10.1007/s12035-014-9029-6 PMC4461562

[13] Kim TH Vemuganti R Effect of sex and age interactions on functional outcome after stroke. CNS Neurosci Ther 2015; 21: 327–336. 25404174 10.1111/cns.12346 PMC6495347

[14] Lopez MS Dempsey RJ Vemuganti R Resveratrol neuroprotection in stroke and traumatic CNS injury. Neurochem Int 2015; 89: 75–82. 26277384 10.1016/j.neuint.2015.08.009 PMC4587342

[15] Dias V Junn E Mouradian MM The role of oxidative stress in Parkinson's disease. J Parkinsons Dis 2013; 3: 461–491. 24252804 10.3233/JPD-130230 PMC4135313

[16] Pacheco C Aguayo LG Opazo C An extracellular mechanism that can explain the neurotoxic effects of alpha-synuclein aggregates in the brain. Front Physiol 2012; 3: 297. 22934048 10.3389/fphys.2012.00297 PMC3429068

[17] Dauer W Przedborski S Parkinson's disease: Mechanisms and models. Neuron 2003; 39: 889–909. 12971891 10.1016/s0896-6273(03)00568-3

[18] Rodriguez-Grande B Blackabey V Gittens B Loss of substance P and inflammation precede delayed neurodegeneration in the substantia nigra after cerebral ischemia. Brain Behav Immun 2013; 29: 51–61. 23232501 10.1016/j.bbi.2012.11.017

[19] Zhao HQ Li FF Wang Z A comparative study of the amount of alpha-synuclein in ischemic stroke and Parkinson's disease. Neurol Sci 2016; 37: 749–754. 26829934 10.1007/s10072-016-2485-1

[20] Benamer HT Grosset DG Vascular parkinsonism: A clinical review. Eur Neurol 2009; 61: 11–15. 18948694 10.1159/000165343

[21] Demirkiran M Bozdemir H Sarica Y Vascular parkinsonism: A distinct, heterogeneous clinical entity. Acta Neurol Scand 2001; 104: 63–67. 11493219 10.1034/j.1600-0404.2001.104002063.x

[22] Murrow RW Schweiger GD Kepes JJ Parkinsonism due to a basal ganglia lacunar state: Clinicopathologic correlation. Neurology 1990; 40: 897–900. 2345612 10.1212/wnl.40.6.897

[23] Hughes AJ Daniel SE Kilford L Accuracy of clinical diagnosis of idiopathic Parkinson's disease: A clinico-pathological study of 100 cases. J Neurol Neurosurg Psychiatry 1992; 55: 181–184. 1564476 10.1136/jnnp.55.3.181 PMC1014720

[24] Hoehn MM Yahr MD Parkinsonism: onset, progression and mortality. Neurology 1967; 17: 427–442. 6067254 10.1212/wnl.17.5.427

[25] Levine RL Jones JC Bee N Stroke and Parkinson's disease. Stroke 1992; 23: 839–842. 1595102 10.1161/01.str.23.6.839

[26] Spillantini MG Schmidt ML Lee VM Alpha-synuclein in Lewy bodies. Nature 1997; 388: 839–840. 9278044 10.1038/42166

[27] Mehta SL Manhas N Raghubir R Molecular targets in cerebral ischemia for developing novel therapeutics. Brain Res Rev 2007; 54: 34–66. 17222914 10.1016/j.brainresrev.2006.11.003

[28] Bartels T Ahlstrom LS Leftin A The N-terminus of the intrinsically disordered protein alpha-synuclein triggers membrane binding and helix folding. Biophys J 2010; 99: 2116–2124. 20923645 10.1016/j.bpj.2010.06.035 PMC3042581

[29] Bertoncini CW Jung YS Fernandez CO Release of long-range tertiary interactions potentiates aggregation of natively unstructured alpha-synuclein. Proc Natl Acad Sci U S A 2005; 102: 1430–1435. 15671169 10.1073/pnas.0407146102 PMC547830

[30] Dedmon MM Lindorff-Larsen K Christodoulou J Mapping long-range interactions in alpha-synuclein using spin-label NMR and ensemble molecular dynamics simulations. J Am Chem Soc 2005; 127: 476–477. 15643843 10.1021/ja044834j

[31] Hejjaoui M Butterfield S Fauvet B Elucidating the role of C-terminal post-translational modifications using protein semisynthesis strategies: Alpha-synuclein phosphorylation at tyrosine 125. J Am Chem Soc 2012; 134: 5196–5210. 22339654 10.1021/ja210866j PMC3592575

[32] Li J Uversky VN Fink AL Effect of familial Parkinson's disease point mutations A30P and A53T on the structural properties, aggregation, and fibrillation of human alpha-synuclein. Biochemistry 2001; 40: 11604–11613. 11560511 10.1021/bi010616g

[33] Chavarria C Souza JM Oxidation and nitration of alpha-synuclein and their implications in neurodegenerative diseases. Arch Biochem Biophys 2013; 533: 25–32. 23454347 10.1016/j.abb.2013.02.009

[34] Proukakis C Dudzik CG Brier T A novel alpha-synuclein missense mutation in Parkinson disease. Neurology 2013; 80: 1062–1064. 23427326 10.1212/WNL.0b013e31828727ba PMC3653201

[35] Lesage S Anheim M Letournel F G51D alpha-synuclein mutation causes a novel parkinsonian-pyramidal syndrome. Ann Neurol 2013; 73: 459–471. 23526723 10.1002/ana.23894

[36] Kowall NW Hantraye P Brouillet E MPTP induces alpha-synuclein aggregation in the substantia nigra of baboons. Neuroreport 2000; 11: 211–213. 10683860 10.1097/00001756-200001170-00041

[37] Narhi L Wood SJ Steavenson S Both familial Parkinson's disease mutations accelerate alpha-synuclein aggregation. J Biol Chem 1999; 274: 9843–9846. 10092675 10.1074/jbc.274.14.9843

[38] Conway KA Harper JD Lansbury PT Accelerated in vitro fibril formation by a mutant alpha-synuclein linked to early-onset Parkinson disease. Nat Med 1998; 4: 1318–1320. 9809558 10.1038/3311

[39] Winner B Jappelli R Maji SK In vivo demonstration that alpha-synuclein oligomers are toxic. Proc Natl Acad Sci U S A 2011; 108: 4194–4199. 21325059 10.1073/pnas.1100976108 PMC3053976

[40] Outeiro TF Putcha P Tetzlaff JE Formation of toxic oligomeric alpha-synuclein species in living cells. PLoS One 2008; 3: e1867. 18382657 10.1371/journal.pone.0001867 PMC2270899

[41] Danzer KM Haasen D Karow AR Different species of alpha-synuclein oligomers induce calcium influx and seeding. J Neurosci 2007; 27: 9220–9232. 17715357 10.1523/JNEUROSCI.2617-07.2007 PMC6672196

[42] Valente EM Abou-Sleiman PM Caputo V Hereditary early-onset Parkinson's disease caused by mutations in PINK1. Science 2004; 30467: 1158–1160. 10.1126/science.1096284 15087508

[43] Ding TT Lee SJ Rochet JC Annular alpha-synuclein protofibrils are produced when spherical protofibrils are incubated in solution or bound to brain-derived membranes. Biochemistry 2002; 41: 10209–10217. 12162735 10.1021/bi020139h

[44] Volles MJ Lee SJ Rochet JC Vesicle permeabilization by protofibrillar alpha-synuclein: Implications for the pathogenesis and treatment of Parkinson's disease. Biochemistry 2001; 40: 7812–7819. 11425308 10.1021/bi0102398

[45] Lashuel HA Lansbury PT Jr Are amyloid diseases caused by protein aggregates that mimic bacterial pore-forming toxins? Q Rev Biophys 2006; 39: 167–201. 16978447 10.1017/S0033583506004422

[46] Stefanovic AN Stockl MT Claessens MM alpha-Synuclein oligomers distinctively permeabilize complex model membranes. FEBS J 2014; 281: 2838–2850. 24767583 10.1111/febs.12824

[47] van Rooijen BD Claessens MM Subramaniam V Membrane permeabilization by oligomeric alpha-synuclein: In search of the mechanism. PLoS One 2010; 5: e14292. 21179192 10.1371/journal.pone.0014292 PMC3001441

[48] Wang C Liu L Zhang L Redox reactions of the alpha-synuclein-Cu+) complex and their effects on neuronal cell viability. Biochemistry 2010; 49: 8134–8142. 20701279 10.1021/bi1010909 PMC2939719

[49] Binolfi A Lamberto GR Duran R Site-specific interactions of Cu(II) with alpha and beta-synuclein: Bridging the molecular gap between metal binding and aggregation. J Am Chem Soc 2008; 130: 11801–11812. 18693689 10.1021/ja803494v

[50] Wang X Moualla D Wright JA Copper binding regulates intracellular alpha-synuclein localisation, aggregation and toxicity. J Neurochem 2010; 113: 704–714. 20141569 10.1111/j.1471-4159.2010.06638.x

[51] Wright JA Wang X Brown DR Unique copper-induced oligomers mediate alpha-synuclein toxicity. FASEB J 2009; 23: 2384–2393. 19325037 10.1096/fj.09-130039

[52] Lindholm D Wootz H Korhonen L ER stress and neurodegenerative diseases. Cell Death Differ 2006; 13: 385–392. 16397584 10.1038/sj.cdd.4401778

[53] Hoozemans JJ van Haastert ES Eikelenboom P Activation of the unfolded protein response in Parkinson's disease. Biochem Biophys Res Commun 2007; 354: 707–711. 17254549 10.1016/j.bbrc.2007.01.043

[54] Colla E Jensen PH Pletnikova O Accumulation of toxic alpha-synuclein oligomer within endoplasmic reticulum occurs in alpha-synucleinopathy in vivo. J Neurosci 2012; 32: 3301–3305. 22399752 10.1523/JNEUROSCI.5368-11.2012 PMC3548448

[55] Smith WW Jiang H Pei Z Endoplasmic reticulum stress and mitochondrial cell death pathways mediate A53T mutant alpha-synuclein-induced toxicity. Hum Mol Genet 2005; 14: 3801–3811. 16239241 10.1093/hmg/ddi396

[56] Fellner L Irschick R Schanda K Toll-like receptor 4 is required for alpha-synuclein dependent activation of microglia and astroglia. Glia 2013; 61: 349–360. 23108585 10.1002/glia.22437 PMC3568908

[57] Zhang W Wang T Pei Z Aggregated alpha-synuclein activates microglia: A process leading to disease progression in Parkinson's disease. FASEB J 2005; 19: 533–542. 15791003 10.1096/fj.04-2751com

[58] Colasanti T Vomero M Alessandri C Role of alpha-synuclein in autophagy modulation of primary human T lymphocytes. Cell Death Dis 2014; 5: e1265. 24874737 10.1038/cddis.2014.211 PMC4047919

[59] Lee HJ Khoshaghideh F Patel S Clearance of alpha-synuclein oligomeric intermediates via the lysosomal degradation pathway. J Neurosci 2004; 24: 1888–1896. 14985429 10.1523/JNEUROSCI.3809-03.2004 PMC6730405

[60] Chen H Chan DC Mitochondrial dynamics–fusion, fission, movement, and mitophagy–in neurodegenerative diseases. Hum Mol Genet 2009; 18: R169–R176. 19808793 10.1093/hmg/ddp326 PMC2758711

[61] Reeve AK Ludtmann MH Angelova PR Aggregated alpha-synuclein and complex I deficiency: Exploration of their relationship in differentiated neurons. Cell Death Dis 2015; 6: e1820. 26181201 10.1038/cddis.2015.166 PMC4650719

[62] Chinta SJ Mallajosyula JK Rane A Mitochondrial alpha-synuclein accumulation impairs complex I function in dopaminergic neurons and results in increased mitophagy in vivo. Neurosci Lett 2010; 486: 235–239. 20887775 10.1016/j.neulet.2010.09.061 PMC2967673

[63] Devi L Raghavendran V Prabhu BM Mitochondrial import and accumulation of alpha-synuclein impair complex I in human dopaminergic neuronal cultures and Parkinson disease brain. J Biol Chem 2008; 283: 9089–9100. 18245082 10.1074/jbc.M710012200 PMC2431021

[64] Poon HF Frasier M Shreve N Mitochondrial associated metabolic proteins are selectively oxidized in A30P alpha-synuclein transgenic mice – A model of familial Parkinson's disease. Neurobiol Dis 2005; 18: 492–498. 15755676 10.1016/j.nbd.2004.12.009

[65] Gui YX Wang XY Kang WY Extracellular signal-regulated kinase is involved in alpha-synuclein-induced mitochondrial dynamic disorders by regulating dynamin-like protein 1. Neurobiol Aging 2012; 33: 2841–2854. 22445325 10.1016/j.neurobiolaging.2012.02.001

[66] Tanaka Y Engelender S Igarashi S Inducible expression of mutant alpha-synuclein decreases proteasome activity and increases sensitivity to mitochondria-dependent apoptosis. Hum Mol Genet 2001; 10: 919–926. 11309365 10.1093/hmg/10.9.919

[67] Zhu M Li W Lu C Role of alpha-synuclein protein levels in mitochondrial morphology and cell survival in cell lines. PLoS One 2012; 7: e36377. 22558453 10.1371/journal.pone.0036377 PMC3338674

[68] Schmid AW Fauvet B Moniatte M Alpha-synuclein post-translational modifications as potential biomarkers for Parkinson disease and other synucleinopathies. Mol Cell Proteomics 2013; 12: 3543–3558. 23966418 10.1074/mcp.R113.032730 PMC3861707

[69] Iwatsubo T Aggregation of alpha-synuclein in the pathogenesis of Parkinson's disease. J Neurol 2003; (250 Suppl 3): III11–14. 14579119 10.1007/s00415-003-1303-x

[70] Fujiwara H Hasegawa M Dohmae N alpha-Synuclein is phosphorylated in synucleinopathy lesions. Nat Cell Biol 2002; 4: 160–164. 11813001 10.1038/ncb748

[71] Chen L Feany MB Alpha-synuclein phosphorylation controls neurotoxicity and inclusion formation in a Drosophila model of Parkinson disease. Nat Neurosci 2005; 8: 657–663. 15834418 10.1038/nn1443

[72] Sato H Arawaka S Hara S Authentically phosphorylated alpha-synuclein at Ser129 accelerates neurodegeneration in a rat model of familial Parkinson's disease. J Neurosci 2011; 31: 16884–16894. 22090514 10.1523/JNEUROSCI.3967-11.2011 PMC6633319

[73] Oueslati A Schneider BL Aebischer P Polo-like kinase 2 regulates selective autophagic alpha-synuclein clearance and suppresses its toxicity in vivo. Proc Natl Acad Sci U S A 2013; 110: E3945–E3954. 23983262 10.1073/pnas.1309991110 PMC3799334

[74] Wang W Perovic I Chittuluru J A soluble alpha-synuclein construct forms a dynamic tetramer. Proc Natl Acad Sci U S A 2011; 108: 17797–1802. 22006323 10.1073/pnas.1113260108 PMC3203798

[75] Bartels T Choi JG Selkoe DJ Alpha-synuclein occurs physiologically as a helically folded tetramer that resists aggregation. Nature 2011; 477: 107–110. 21841800 10.1038/nature10324 PMC3166366

[76] Dettmer U Newman AJ Soldner F Parkinson-causing alpha-synuclein missense mutations shift native tetramers to monomers as a mechanism for disease initiation. Nat Commun 2015; 6: 7314. 26076669 10.1038/ncomms8314 PMC4490410

[77] Dettmer U Newman AJ von Saucken VE KTKEGV repeat motifs are key mediators of normal alpha-synuclein tetramerization: Their mutation causes excess monomers and neurotoxicity. Proc Natl Acad Sci U S A 2015; 112: 9596–9601. 26153422 10.1073/pnas.1505953112 PMC4534262

[78] Burre J Vivona S Diao J Properties of native brain alpha-synuclein. Nature 2013; 49845 : E4-6; discussion E6-7. 10.1038/nature12125 PMC4255827 23765500

[79] Fauvet B Mbefo MK Fares MB alpha-Synuclein in central nervous system and from erythrocytes, mammalian cells, and Escherichia coli exists predominantly as disordered monomer. J Biol Chem 2012; 287: 15345–15364. 22315227 10.1074/jbc.M111.318949 PMC3346117

[80] Doxakis E Post-transcriptional regulation of alpha-synuclein expression by mir-7 and mir-153. J Biol Chem 2010; 285: 12726–12734. 20106983 10.1074/jbc.M109.086827 PMC2857101

[81] Junn E Lee KW Jeong BS Repression of alpha-synuclein expression and toxicity by microRNA-7. Proc Natl Acad Sci U S A 2009; 106: 13052–13057. 19628698 10.1073/pnas.0906277106 PMC2722353

[82] Thome AD Harms AS Volpicelli-Daley LA microRNA-155 regulates alpha-synuclein-induced inflammatory responses in models of Parkinson disease. J Neurosci 2016; 36: 2383–2390. 26911687 10.1523/JNEUROSCI.3900-15.2016 PMC4764660

[83] Qiu C Xu W Winblad B Vascular risk profiles for dementia and Alzheimer's disease in very old people: A population-based longitudinal study. J Alzheimers Dis 2010; 20: 293–300. 20164587 10.3233/JAD-2010-1361

[84] Ishimaru H Ueda K Takahashi A Changes in presynaptic protein NACP/alpha-synuclein in an ischemic gerbil hippocampus. Brain Res 1998; 788: 311–314. 9555070 10.1016/s0006-8993(98)00033-x

[85] Polymeropoulos MH Lavedan C Leroy E Mutation in the alpha-synuclein gene identified in families with Parkinson's disease. Science 1997; 276: 2045–2047. 9197268 10.1126/science.276.5321.2045

[86] Hu X Rea HC Wiktorowicz JE Proteomic analysis of hypoxia/ischemia-induced alteration of cortical development and dopamine neurotransmission in neonatal rat. J Proteome Res 2006; 5: 2396–2404. 16944952 10.1021/pr060209x PMC3128998

[87] Yoon DK Hwang IK Yoo KY Comparison of alpha-synuclein immunoreactivity and protein levels in ischemic hippocampal CA1 region between adult and aged gerbils and correlation with Cu,Zn-superoxide dismutase. Neurosci Res 2006; 55: 434–441. 16759729 10.1016/j.neures.2006.04.014

[88] Golovko MY Murphy EJ Brain prostaglandin formation is increased by alpha-synuclein gene-ablation during global ischemia. Neurosci Lett 2008; 432: 243–247. 18226447 10.1016/j.neulet.2007.12.031 PMC2274002

[89] Joo M Sadikot RT PGD synthase and PGD2 in immune resposne. Mediators Inflamm 2012; 2012: 503128. 22791937 10.1155/2012/503128 PMC3389719

[90] Saleem S Zhuang H de Brum-Fernandes AJ PGD DP1 receptor protects brain from ischemia-reperfusion injury. Eur J Neurosci 2007; 26: 73–78. 17573924 10.1111/j.1460-9568.2007.05627.x PMC2386988

[91] Unal-Cevik I Gursoy-Ozdemir Y Yemisci M Alpha-synuclein aggregation induced by brief ischemia negatively impacts neuronal survival in vivo: a study in [A30P]alpha-synuclein transgenic mouse. J Cereb Blood Flow Metab 2011; 31: 913–923. 20877387 10.1038/jcbfm.2010.170 PMC3063624

[92] Kim T Mehta SL Kaimal B Poststroke induction of alpha-synuclein mediates ischemic brain damage. J Neurosci 2016; 36: 7055–7065. 27358461 10.1523/JNEUROSCI.1241-16.2016 PMC4994709

[93] Dong Z Ferger B Paterna JC Dopamine-dependent neurodegeneration in rats induced by viral vector-mediated overexpression of the parkin target protein, CDCrel-1. Proc Natl Acad Sci U S A 2003; 100: 12438–12443. 14530399 10.1073/pnas.2132992100 PMC218776

[94] Zhang Y Gao J Chung KK Parkin functions as an E2-dependent ubiquitin-protein ligase and promotes the degradation of the synaptic vesicle-associated protein, CDCrel-1. Proc Natl Acad Sci U S A 2000; 97: 13354–13359. 11078524 10.1073/pnas.240347797 PMC27228

[95] Yang Y Nishimura I Imai Y Parkin suppresses dopaminergic neuron-selective neurotoxicity induced by Pael-R in Drosophila. Neuron 2003; 37: 911–924. 12670421 10.1016/s0896-6273(03)00143-0

[96] Chung KK Zhang Y Lim KL Parkin ubiquitinates the alpha-synuclein-interacting protein, synphilin-1: Implications for Lewy-body formation in Parkinson disease. Nat Med 2001; 7: 1144–1150. 11590439 10.1038/nm1001-1144

[97] Lim KL Chew KC Tan JM Parkin mediates nonclassical, proteasomal-independent ubiquitination of synphilin-1: Implications for Lewy body formation. J Neurosci 2005; 25: 2002–2009. 15728840 10.1523/JNEUROSCI.4474-04.2005 PMC6726069

[98] Petrucelli L O'Farrell C Lockhart PJ Parkin protects against the toxicity associated with mutant alpha-synuclein: Proteasome dysfunction selectively affects catecholaminergic neurons. Neuron 2002; 36: 1007–1019. 12495618 10.1016/s0896-6273(02)01125-x

[99] Staropoli JF McDermott C Martinat C Parkin is a component of an SCF-like ubiquitin ligase complex and protects postmitotic neurons from kainate excitotoxicity. Neuron 2003; 37: 735–749. 12628165 10.1016/s0896-6273(03)00084-9

[100] Lucking CB Durr A Bonifati V Association between early-onset Parkinson's disease and mutations in the parkin gene. N Engl J Med 2000; 342: 1560–1567. 10824074 10.1056/NEJM200005253422103

[101] Torrelles JB DesJardin LE MacNeil J Inactivation of Mycobacterium tuberculosis mannosyltransferase pimB reduces the cell wall lipoarabinomannan and lipomannan content and increases the rate of bacterial-induced human macrophage cell death. Glycobiology 2009; 19: 743–755. 19318518 10.1093/glycob/cwp042 PMC2688391

[102] Wang C Ko HS Thomas B Stress-induced alterations in parkin solubility promote parkin aggregation and compromise parkin's protective function. Hum Mol Genet 2005; 14: 3885–3897. 16278233 10.1093/hmg/ddi413

[103] Ko HS Kim SW Sriram SR Identification of far upstream element-binding protein-1 as an authentic Parkin substrate. J Biol Chem 2006; 281: 16193–16196. 16672220 10.1074/jbc.C600041200

[104] Ko HS von Coelln R Sriram SR Accumulation of the authentic parkin substrate aminoacyl-tRNA synthetase cofactor, p38/JTV-1, leads to catecholaminergic cell death. J Neurosci 2005; 25: 7968–7978. 16135753 10.1523/JNEUROSCI.2172-05.2005 PMC6725452

[105] Ko HS Lee Y Shin JH Phosphorylation by the c-Abl protein tyrosine kinase inhibits parkin's ubiquitination and protective function. Proc Natl Acad Sci U S A 2010; 107: 16691–16696. 20823226 10.1073/pnas.1006083107 PMC2944759

[106] Brahmachari S Ge P Lee SH Activation of tyrosine kinase c-Abl contributes to alpha-synuclein-induced neurodegeneration. J Clin Invest 2016; 126: 2970–2988. 27348587 10.1172/JCI85456 PMC4966315

[107] Shin JH Ko HS Kang H PARIS (ZNF74 repression of PGC-1alpha contributes to neurodegeneration in Parkinson's disease. Cell 2011; 144: 689–702. 21376232 10.1016/j.cell.2011.02.010 PMC3063894

[108] Romani-Aumedes J Canal M Martin-Flores N Parkin loss of function contributes to RTP801 elevation and neurodegeneration in Parkinson's disease. Cell Death Dis 2014; 5: e1364. 25101677 10.1038/cddis.2014.333 PMC4454308

[109] Gandhi S Muqit MM Stanyer L PINK1 protein in normal human brain and Parkinson's disease. Brain 2006; 129: 1720–1731. 16702191 10.1093/brain/awl114

[110] Pilsl A Winklhofer KF Parkin, PINK1 and mitochondrial integrity: Emerging concepts of mitochondrial dysfunction in Parkinson's disease. Acta Neuropathol 2012; 123: 173–188. 22057787 10.1007/s00401-011-0902-3

[111] Corti O Lesage S Brice A What genetics tells us about the causes and mechanisms of Parkinson's disease. Physiol Rev 2011; 91: 1161–1218. 22013209 10.1152/physrev.00022.2010

[112] Oliveras-Salva M Macchi F Coessens V Alpha-synuclein-induced neurodegeneration is exacerbated in PINK1 knockout mice. Neurobiol Aging 2014; 35: 2625–2636. 25037286 10.1016/j.neurobiolaging.2014.04.032

[113] Liu W Vives-Bauza C Acin-Perez R PINK1 defect causes mitochondrial dysfunction, proteasomal deficit and alpha-synuclein aggregation in cell culture models of Parkinson's disease. PLoS One 2009; 4: e4597. 19242547 10.1371/journal.pone.0004597 PMC2644779

[114] Todd AM Staveley BE Pink1 suppresses alpha-synuclein-induced phenotypes in a Drosophila model of Parkinson's disease. Genome 2008; 51: 1040–1046. 19088817 10.1139/G08-085

[115] Pridgeon JW Olzmann JA Chin LS PINK1 protects against oxidative stress by phosphorylating mitochondrial chaperone TRAP1. PLoS Biol 2007; 5: e172. 17579517 10.1371/journal.pbio.0050172 PMC1892574

[116] Zhang L Karsten P Hamm S TRAP1 rescues PINK1 loss-of-function phenotypes. Hum Mol Genet 2013; 22: 2829–2841. 23525905 10.1093/hmg/ddt132 PMC3690968

[117] Kim I Rodriguez-Enriquez S Lemasters JJ Selective degradation of mitochondria by mitophagy. Arch Biochem Biophys 2007; 462: 245–253. 17475204 10.1016/j.abb.2007.03.034 PMC2756107

[118] Matsuda N Sato S Shiba K PINK1 stabilized by mitochondrial depolarization recruits Parkin to damaged mitochondria and activates latent Parkin for mitophagy. J Cell Biol 2010; 189: 211–221. 20404107 10.1083/jcb.200910140 PMC2856912

[119] Narendra D Kane LA Hauser DN p62/SQSTM1 is required for Parkin-induced mitochondrial clustering but not mitophagy; VDAC1 is dispensable for both. Autophagy 2010; 6: 1090–1106. 20890124 10.4161/auto.6.8.13426 PMC3359490

[120] Narendra D Tanaka A Suen DF Parkin is recruited selectively to impaired mitochondria and promotes their autophagy. J Cell Biol 2008; 183: 795–803. 19029340 10.1083/jcb.200809125 PMC2592826

[121] Koyano F Okatsu K Kosako H Ubiquitin is phosphorylated by PINK1 to activate parkin. Nature 2014; 510: 162–166. 24784582 10.1038/nature13392

[122] Chen Y Dorn GW 2nd PINK1-phosphorylated mitofusin 2 is a Parkin receptor for culling damaged mitochondria. Science 2013; 340: 471–475. 23620051 10.1126/science.1231031 PMC3774525

[123] Gegg ME Cooper JM Chau KY Mitofusin 1 and mitofusin 2 are ubiquitinated in a PINK1/parkin-dependent manner upon induction of mitophagy. Hum Mol Genet 2010; 19: 4861–4870. 20871098 10.1093/hmg/ddq419 PMC3583518

[124] Glauser L Sonnay S Stafa K Parkin promotes the ubiquitination and degradation of the mitochondrial fusion factor mitofusin 1. J Neurochem 2011; 118: 636–645. 21615408 10.1111/j.1471-4159.2011.07318.x

[125] Lazarou M Sliter DA Kane LA The ubiquitin kinase PINK1 recruits autophagy receptors to induce mitophagy. Nature 2015; 524: 309–314. 26266977 10.1038/nature14893 PMC5018156

[126] Zhao Y Chen F Chen S The Parkinson's disease-associated gene PINK1 protects neurons from ischemic damage by decreasing mitochondrial translocation of the fission promoter Drp1. J Neurochem 2013; 127: 711–722. 23772688 10.1111/jnc.12340

[127] Huang Y Chen HJ Zhu JH [Effects of PINK1 gene on cell apoptosis and cell autophagy in neonatal mice with hypoxic-ischemic brain damage]. Zhongguo Dang Dai Er Ke Za Zhi 2016; 18: 263–269. 26975827 10.7499/j.issn.1008-8830.2016.03.015 PMC7389992

[128] Chen SD Lin TK Yang DI Roles of PTEN-induced putative kinase 1 and dynamin-related protein 1 in transient global ischemia-induced hippocampal neuronal injury. Biochem Biophys Res Commun 2015; 460: 397–403. 25791474 10.1016/j.bbrc.2015.03.045

[129] Mengesdorf T Jensen PH Mies G Down-regulation of parkin protein in transient focal cerebral ischemia: A link between stroke and degenerative disease? Proc Natl Acad Sci U S A 2002; 99: 15042–15047. 12415119 10.1073/pnas.232588799 PMC137541

[130] Zhang X Yuan Y Jiang L Endoplasmic reticulum stress induced by tunicamycin and thapsigargin protects against transient ischemic brain injury: Involvement of PARK2-dependent mitophagy. Autophagy 2014; 10: 1801–1813. 25126734 10.4161/auto.32136 PMC4198364

[131] Zhang X Yan H Yuan Y Cerebral ischemia-reperfusion-induced autophagy protects against neuronal injury by mitochondrial clearance. Autophagy 2013; 9: 1321–1333. 23800795 10.4161/auto.25132

[132] Uzdensky A Demyanenko S Fedorenko G Protein profile and morphological alterations in penumbra after focal photothrombotic infarction in the rat cerebral cortex. Mol Neurobiol. Epub ahead of print 21 June 2016 DOI:10.1007/s12035-016-9964-5. 10.1007/s12035-016-9964-5 27324898

[133] Demyanenko SV Panchenko SN Uzdensky AB Expression of neuronal and signaling proteins in penumbra around a photothrombotic infarction core in rat cerebral cortex. Biochemistry 2015; 80: 790–799. 26531025 10.1134/S0006297915060152

[134] Zhou M Xia ZY Lei SQ Role of mitophagy regulated by Parkin/DJ-1 in remote ischemic postconditioning-induced mitigation of focal cerebral ischemia-reperfusion. Eur Rev Med Pharmacol Sci 2015; 19: 4866–4871. 26744879

[135] Zhang L Shimoji M Thomas B Mitochondrial localization of the Parkinson's disease related protein DJ-1: Implications for pathogenesis. Hum Mol Genet 2005; 14: 2063–2073. 15944198 10.1093/hmg/ddi211

[136] Wilson MA Collins JL Hod Y The 1.1-A resolution crystal structure of DJ-1, the protein mutated in autosomal recessive early onset Parkinson's disease. Proc Natl Acad Sci U S A 2003; 100: 9256–9261. 12855764 10.1073/pnas.1133288100 PMC170905

[137] Nagakubo D Taira T Kitaura H DJ-1, a novel oncogene which transforms mouse NIH3T3 cells in cooperation with ras. Biochem Biophys Res Commun 1997; 231: 509–513. 9070310 10.1006/bbrc.1997.6132

[138] Inden M Taira T Kitamura Y PARK7 DJ-1 protects against degeneration of nigral dopaminergic neurons in Parkinson's disease rat model. Neurobiol Dis 2006; 24: 144–158. 16860563 10.1016/j.nbd.2006.06.004

[139] Taira T Saito Y Niki T DJ-1 has a role in antioxidative stress to prevent cell death. EMBO Rep 2004; 5: 213–218. 14749723 10.1038/sj.embor.7400074 PMC1298985

[140] Yokota T Sugawara K Ito K Down regulation of DJ-1 enhances cell death by oxidative stress, ER stress, and proteasome inhibition. Biochem Biophys Res Commun 2003; 312: 1342–1348. 14652021 10.1016/j.bbrc.2003.11.056

[141] Hayashi T Ishimori C Takahashi-Niki K DJ-1 binds to mitochondrial complex I and maintains its activity. Biochem Biophys Res Commun 2009; 390: 667–672. 19822128 10.1016/j.bbrc.2009.10.025

[142] Giaime E Yamaguchi H Gautier CA Loss of DJ-1 does not affect mitochondrial respiration but increases ROS production and mitochondrial permeability transition pore opening. PLoS One 2012; 7: e40501. 22792356 10.1371/journal.pone.0040501 PMC3392228

[143] Irrcher I Aleyasin H Seifert EL Loss of the Parkinson's disease-linked gene DJ-1 perturbs mitochondrial dynamics. Hum Mol Genet 2010; 19: 3734–3746. 20639397 10.1093/hmg/ddq288

[144] Krebiehl G Ruckerbauer S Burbulla LF Reduced basal autophagy and impaired mitochondrial dynamics due to loss of Parkinson's disease-associated protein DJ-1. PLoS One 2010; 5: e9367. 20186336 10.1371/journal.pone.0009367 PMC2826413

[145] Thomas KJ McCoy MK Blackinton J DJ-1 acts in parallel to the PINK1/parkin pathway to control mitochondrial function and autophagy. Hum Mol Genet 2011; 20: 40–50. 20940149 10.1093/hmg/ddq430 PMC3000675

[146] Choi J Sullards MC Olzmann JA Oxidative damage of DJ-1 is linked to sporadic Parkinson and Alzheimer diseases. J Biol Chem 2006; 281: 10816–10824. 16517609 10.1074/jbc.M509079200 PMC1850953

[147] Bandopadhyay R Kingsbury AE Cookson MR The expression of DJ-1 (PARK in normal human CNS and idiopathic Parkinson's disease. Brain 2004; 127: 420–430. 14662519 10.1093/brain/awh054

[148] Blackinton J Lakshminarasimhan M Thomas KJ Formation of a stabilized cysteine sulfinic acid is critical for the mitochondrial function of the parkinsonism protein DJ-1. J Biol Chem 2009; 284: 6476–6485. 19124468 10.1074/jbc.M806599200 PMC2649108

[149] Andres-Mateos E Perier C Zhang L DJ-1 gene deletion reveals that DJ-1 is an atypical peroxiredoxin-like peroxidase. Proc Natl Acad Sci U S A 2007; 104: 14807–14812. 17766438 10.1073/pnas.0703219104 PMC1976193

[150] Canet-Aviles RM Wilson MA Miller DW The Parkinson's disease protein DJ-1 is neuroprotective due to cysteine-sulfinic acid-driven mitochondrial localization. Proc Natl Acad Sci U S A 2004; 101: 9103–9108. 15181200 10.1073/pnas.0402959101 PMC428480

[151] Bandyopadhyay S Cookson MR Evolutionary and functional relationships within the DJ1 superfamily. BMC Evol Biol 2004; 4: 6. 15070401 10.1186/1471-2148-4-6 PMC385224

[152] Kinumi T Kimata J Taira T Cysteine-106 of DJ-1 is the most sensitive cysteine residue to hydrogen peroxide-mediated oxidation in vivo in human umbilical vein endothelial cells. Biochem Biophys Res Commun 2004; 317: 722–728. 15081400 10.1016/j.bbrc.2004.03.110

[153] Shinbo Y Niki T Taira T Proper SUMO-1 conjugation is essential to DJ-1 to exert its full activities. Cell Death Differ 2006; 13: 96–108. 15976810 10.1038/sj.cdd.4401704

[154] Fan J Ren H Fei E Sumoylation is critical for DJ-1 to repress p53 transcriptional activity. FEBS Lett 2008; 582: 1151–1156. 18339323 10.1016/j.febslet.2008.03.003

[155] Ito G Ariga H Nakagawa Y Roles of distinct cysteine residues in S-nitrosylation and dimerization of DJ-1. Biochem Biophys Res Commun 2006; 339: 667–72. 16316629 10.1016/j.bbrc.2005.11.058

[156] Choi MS Nakamura T Cho SJ Transnitrosylation from DJ-1 to PTEN attenuates neuronal cell death in parkinson's disease models. J Neurosci 2014; 34: 15123–15131. 25378175 10.1523/JNEUROSCI.4751-13.2014 PMC4220036

[157] Duan X Kelsen SG Merali S Proteomic analysis of oxidative stress-responsive proteins in human pneumocytes: Insight into the regulation of DJ-1 expression. J Proteome Res 2008; 7: 4955–4961. 18817430 10.1021/pr800295j

[158] Zhou W Zhu M Wilson MA The oxidation state of DJ-1 regulates its chaperone activity toward alpha-synuclein. J Mol Biol 2006; 356: 1036–1048. 16403519 10.1016/j.jmb.2005.12.030

[159] Shendelman S Jonason A Martinat C DJ-1 is a redox-dependent molecular chaperone that inhibits alpha-synuclein aggregate formation. PLoS Biol 2004; 2: e362. 15502874 10.1371/journal.pbio.0020362 PMC521177

[160] Zondler L Miller-Fleming L Repici M DJ-1 interactions with alpha-synuclein attenuate aggregation and cellular toxicity in models of Parkinson's disease. Cell Death Dis 2014; 5: e1350. 25058424 10.1038/cddis.2014.307 PMC4123098

[161] Girotto S Cendron L Bisaglia M DJ-1 is a copper chaperone acting on SOD1 activation. J Biol Chem 2014; 289: 10887–10899. 24567322 10.1074/jbc.M113.535112 PMC4036200

[162] Kato I Maita H Takahashi-Niki K Oxidized DJ-1 inhibits p53 by sequestering p53 from promoters in a DNA-binding affinity-dependent manner. Mol Cell Biol 2013; 33: 340–359. 23149933 10.1128/MCB.01350-12 PMC3554126

[163] Shinbo Y Taira T Niki T DJ-1 restores p53 transcription activity inhibited by Topors/p53BP3. Int J Oncol 2005; 26: 641–648. 15703819

[164] Im JY Lee KW Woo JM DJ-1 induces thioredoxin 1 expression through the Nrf2 pathway. Hum Mol Genet 2012; 21: 3013–3024. 22492997 10.1093/hmg/dds131 PMC3373246

[165] Clements CM McNally RS Conti BJ DJ-1, a cancer- and Parkinson's disease-associated protein, stabilizes the antioxidant transcriptional master regulator Nrf2. Proc Natl Acad Sci U S A 2006; 103: 15091–15096. 17015834 10.1073/pnas.0607260103 PMC1586179

[166] Zhong N Kim CY Rizzu P DJ-1 transcriptionally up-regulates the human tyrosine hydroxylase by inhibiting the sumoylation of pyrimidine tract-binding protein-associated splicing factor. J Biol Chem 2006; 281: 20940–20948. 16731528 10.1074/jbc.M601935200

[167] Mitsugi H Niki T Takahashi-Niki K Identification of the recognition sequence and target proteins for DJ-1 protease. FEBS Lett 2013; 587: 2493–2499. 23831022 10.1016/j.febslet.2013.06.032

[168] Chen J Li L Chin LS Parkinson disease protein DJ-1 converts from a zymogen to a protease by carboxyl-terminal cleavage. Hum Mol Genet 2010; 19: 2395–2408. 20304780 10.1093/hmg/ddq113 PMC2876885

[169] Hao LY Giasson BI Bonini NM DJ-1 is critical for mitochondrial function and rescues PINK1 loss of function. Proc Natl Acad Sci U S A 2010; 107: 9747–9452. 20457924 10.1073/pnas.0911175107 PMC2906840

[170] Kamp F Exner N Lutz AK Inhibition of mitochondrial fusion by alpha-synuclein is rescued by PINK1, Parkin and DJ-1. EMBO J 2010; 29: 3571–3589. 20842103 10.1038/emboj.2010.223 PMC2964170

[171] Yanagisawa D Kitamura Y Inden M DJ-1 protects against neurodegeneration caused by focal cerebral ischemia and reperfusion in rats. J Cereb Blood Flow Metab 2008; 28: 563–578. 17882163 10.1038/sj.jcbfm.9600553

[172] Kitamura Y Watanabe S Taguchi M Neuroprotective effect of a new DJ-1-binding compound against neurodegeneration in Parkinson's disease and stroke model rats. Mol Neurodegener 2011; 6: 48. 21740546 10.1186/1750-1326-6-48 PMC3141555

[173] Yu HH Xu Q Chen HP Stable overexpression of DJ-1 protects H9c2 cells against oxidative stress under a hypoxia condition. Cell Biochem Funct 2013; 31: 643–651. 23281015 10.1002/cbf.2949

[174] Aleyasin H Rousseaux MW Phillips M The Parkinson's disease gene DJ-1 is also a key regulator of stroke-induced damage. Proc Natl Acad Sci U S A 2007; 104: 18748–18753. 18003894 10.1073/pnas.0709379104 PMC2141848

[175] Kaneko Y Tajiri N Shojo H Oxygen-glucose-deprived rat primary neural cells exhibit DJ-1 translocation into healthy mitochondria: A potent stroke therapeutic target. CNS Neurosci Ther 2014; 20: 275–281. 24382215 10.1111/cns.12208 PMC3947479

[176] Kaneko Y Shojo H Burns J DJ-1 ameliorates ischemic cell death in vitro possibly via mitochondrial pathway. Neurobiol Dis 2014; 62: 56–61. 24060818 10.1016/j.nbd.2013.09.007 PMC4083678

[177] Yanagida T Tsushima J Kitamura Y Oxidative stress induction of DJ-1 protein in reactive astrocytes scavenges free radicals and reduces cell injury. Oxid Med Cell Longev 2009; 2: 36–42. 20046643 10.4161/oxim.2.1.7985 PMC2763229

[178] Meulener MC Graves CL Sampathu DM DJ-1 is present in a large molecular complex in human brain tissue and interacts with alpha-synuclein. J Neurochem 2005; 93: 1524–1532. 15935068 10.1111/j.1471-4159.2005.03145.x

[179] Nural H He P Beach T Dissembled DJ-1 high molecular weight complex in cortex mitochondria from Parkinson's disease patients. Mol Neurodegener 2009; 4: 23. 19497122 10.1186/1750-1326-4-23 PMC2704189

[180] Neumann M Muller V Gorner K Pathological properties of the Parkinson's disease-associated protein DJ-1 in alpha-synucleinopathies and tauopathies: Relevance for multiple system atrophy and Pick's disease. Acta Neuropathol 2004; 107: 489–496. 14991385 10.1007/s00401-004-0834-2

[181] Bretaud S Allen C Ingham PW p53-dependent neuronal cell death in a DJ-1-deficient zebrafish model of Parkinson's disease. J Neurochem 2007; 100: 1626–1635. 17166173 10.1111/j.1471-4159.2006.04291.x

[182] Zhang P Lei X Sun Y Regenerative repair of Pifithrin-alpha in cerebral ischemia via VEGF dependent manner. Sci Rep 2016; 6: 26295. 27212231 10.1038/srep26295 PMC4876321

[183] Leker RR Aharonowiz M Greig NH The role of p53-induced apoptosis in cerebral ischemia: Effects of the p53 inhibitor pifithrin alpha. Exp Neurol 2004; 187: 478–486. 15144874 10.1016/j.expneurol.2004.01.030

[184] Culmsee C Zhu X Yu QS A synthetic inhibitor of p53 protects neurons against death induced by ischemic and excitotoxic insults, and amyloid beta-peptide. J Neurochem 2001; 77: 220–228. 11279278 10.1046/j.1471-4159.2001.t01-1-00220.x

[185] Kett LR Dauer WT Leucine-rich repeat kinase 2 for beginners: Six key questions. Cold Spring Harb Perspect Med 2012; 2: a009407. 22393539 10.1101/cshperspect.a009407 PMC3282500

[186] Healy DG Falchi M O'Sullivan SS Phenotype, genotype, and worldwide genetic penetrance of LRRK2-associated Parkinson's disease: A case-control study. Lancet Neurol 2008; 7: 583–590. 18539534 10.1016/S1474-4422(08)70117-0 PMC2832754

[187] Daniels V Vancraenenbroeck R Law BM Greggio E Lobbestael E Gao F Insight into the mode of action of the LRRK2 Y1699C pathogenic mutant. J Neurochem 2011; 116: 304–315. 21073465 10.1111/j.1471-4159.2010.07105.x PMC3005098

[188] Lovitt B Vanderporten EC Sheng Z Differential effects of divalent manganese and magnesium on the kinase activity of leucine-rich repeat kinase 2 (LRRK. Biochemistry 2010; 49: 3092–3100. 20205471 10.1021/bi901726c

[189] Greggio E Jain S Kingsbury A Kinase activity is required for the toxic effects of mutant LRRK2/dardarin. Neurobiol Dis 2006; 23: 329–341. 16750377 10.1016/j.nbd.2006.04.001

[190] Thaler A Ash E Gan-Or Z The LRRK2 G2019S mutation as the cause of Parkinson's disease in Ashkenazi Jews. J Neural Transm 2009; 116: 1473–1482. 19756366 10.1007/s00702-009-0303-0

[191] Lesage S Durr A Tazir M LRRK2 G2019S as a cause of Parkinson's disease in North African Arabs. N Engl J Med 2006; 354: 422–423. 16436781 10.1056/NEJMc055540

[192] Satake W Nakabayashi Y Mizuta I Genome-wide association study identifies common variants at four loci as genetic risk factors for Parkinson's disease. Nat Genet 2009; 41: 1303–1307. 19915576 10.1038/ng.485

[193] Simon-Sanchez J Schulte C Bras JM Genome-wide association study reveals genetic risk underlying Parkinson's disease. Nat Genet 2009; 41: 1308–1312. 19915575 10.1038/ng.487 PMC2787725

[194] Li K Tang BS Liu ZH LRRK2 A419V variant is a risk factor for Parkinson's disease in Asian population. Neurobiol Aging 2015; 36: 2908 e11–e15. 10.1016/j.neurobiolaging.2015.07.012 26234753

[195] Ross OA Wu YR Lee MC Analysis of Lrrk2 R1628P as a risk factor for Parkinson's disease. Ann Neurol 2008; 64: 88–92. 18412265 10.1002/ana.21405

[196] Farrer MJ Stone JT Lin CH Lrrk2 G2385R is an ancestral risk factor for Parkinson's disease in Asia. Parkinsonism Relat Disord 2007; 13: 89–92. 17222580 10.1016/j.parkreldis.2006.12.001

[197] Cookson MR Hardy J Lewis PA Genetic neuropathology of Parkinson's disease. Int J Clin Exp Pathol 2008; 1: 217–231. 18784814 PMC2480564

[198] Rajput A Dickson DW Robinson CA Parkinsonism, Lrrk2 G2019S, and tau neuropathology. Neurology 2006; 67: 1506–1508. 17060589 10.1212/01.wnl.0000240220.33950.0c

[199] Kawakami F Shimada N Ohta E Leucine-rich repeat kinase 2 regulates tau phosphorylation through direct activation of glycogen synthase kinase-3beta. FEBS J 2014; 281: 3–13. 24165324 10.1111/febs.12579

[200] Kawakami F Yabata T Ohta E LRRK2 phosphorylates tubulin-associated tau but not the free molecule: LRRK2-mediated regulation of the tau-tubulin association and neurite outgrowth. PLoS One 2012; 7: e30834. 22303461 10.1371/journal.pone.0030834 PMC3267742

[201] Avila J Gomez de Barreda E Engel T Tau phosphorylation in hippocampus results in toxic gain-of-function. Biochem Soc Trans 2010; 38: 977–980. 20658988 10.1042/BST0380977

[202] Kawakami F Ichikawa T The Role of alpha-Synuclein and LRRK2 in Tau Phosphorylation. Parkinsons Dis 2015; 2015: 734–746. 10.1155/2015/734746 PMC4419261 25977830

[203] Martin L Page G Terro F Tau phosphorylation and neuronal apoptosis induced by the blockade of PP2A preferentially involve GSK3beta. Neurochem Int 2011; 59: 235–250. 21672577 10.1016/j.neuint.2011.05.010

[204] Kawakami F Suzuki M Shimada N Stimulatory effect of alpha-synuclein on the tau-phosphorylation by GSK-3beta. FEBS J 2011; 278: 4895–4904. 21985244 10.1111/j.1742-4658.2011.08389.x

[205] Duka T Duka V Joyce JN Alpha-Synuclein contributes to GSK-3beta-catalyzed Tau phosphorylation in Parkinson's disease models. FASEB J 2009; 23: 2820–2830. 19369384 10.1096/fj.08-120410 PMC2796901

[206] Song B Ao Q Wang Z Phosphorylation of tau protein over time in rats subjected to transient brain ischemia. Neural Regen Res 2013; 8: 3173–3182. 25206638 10.3969/j.issn.1673-5374.2013.34.001 PMC4146185

[207] Wen Y Yang S Liu R Transient cerebral ischemia induces site-specific hyperphosphorylation of tau protein. Brain Res 2004; 1022: 30–38. 15353210 10.1016/j.brainres.2004.05.106

[208] Chen X Liu Y Zhu J GSK-3beta downregulates Nrf2 in cultured cortical neurons and in a rat model of cerebral ischemia-reperfusion. Sci Rep 2016; 6: 20196. 26838164 10.1038/srep20196 PMC4738318

[209] Jo C Gundemir S Pritchard S Nrf2 reduces levels of phosphorylated tau protein by inducing autophagy adaptor protein NDP52. Nat Commun 2014; 5: 3496. 24667209 10.1038/ncomms4496 PMC3990284

